# “Respecting our patients’ choices”: making the organizational decision to participate in voluntary assisted dying provision: findings from semi-structured interviews with a rural community hospice board of management

**DOI:** 10.1186/s12904-022-01051-x

**Published:** 2022-09-16

**Authors:** Kirsten Auret, Terri J. Pikora, Kate Gersbach, Robert J. Donovan

**Affiliations:** 1 Rural Clinical School of Western Australia, University of Western Australia, M701 31 Stirling Terrace, Albany, WA 6330 Australia; 2 Formerly at Albany Community Hospice, WA 6330 Albany, Australia; 3 School of Human Sciences, University of Western Australia, Nedlands, WA 6009 Australia

**Keywords:** Death, Assisted, Death with Dignity, Respect, Hospice Care, Qualitative Research, Health Policy

## Abstract

**Background:**

There is limited literature around how palliative care organizations determine the degree to which they will interface with voluntary assisted dying in jurisdictions where it is legal. The aim of this research was to describe the experience of the board of management of an Australian community-based hospice during their decision-making process around whether to support voluntary assisted dying in the facility, prior to the legislation coming into operation.

**Methods:**

The Board considered this decision over ten meetings in 2020, during which time they received information on the legislation, relevant literature, feedback from workshops which included the community, comment from hospice founders, staff survey results and presentations by clinicians able to discuss the impact of voluntary assisted dying on palliative care services. Members were encouraged to make notes of their own experiences during this time. Following this, semi-structured interviews were conducted with seven of the nine board members. Interviews were audio-recorded and transcribed verbatim and analysed using conventional qualitative content analysis method.

**Results:**

The board members experienced a sense of journey in reaching an overall decision, which was to allow full participation in voluntary assisted dying provision for inpatients. Themes based on the journey motif included: starting from a personal view; moving to a hospice perspective; exploring if voluntary assisted dying can be part of end-of-life care; awareness and assessment of risks to the Hospice; arriving at a common platform to vote on; factors facilitating a safe decision-making journey; and personal impact of the journey.

**Conclusions:**

The group highlighted several facilitators of a successful outcome including having adequate time, the availability of useful resources, sound board processes and a trusting culture. The study may provide support to other healthcare organisations as they face similar decisions triggered by legislative change.

**Supplementary Information:**

The online version contains supplementary material available at 10.1186/s12904-022-01051-x.

## Introduction

The Voluntary Assisted Dying (VAD) Act [1] was passed in Western Australia (WA) in 2019 coming into operation on 1 July 2021. As occurred in Victoria [2], the first Australian State in which VAD had been legalised, there was an 18-month implementation period during which government encouraged healthcare services in WA to consider how VAD may be implemented within the context of existing care options available to people at the end-of-life. During this period, organisations were challenged to make policy decisions about how they would support their patients if requesting access to VAD. Potential options ranged from organisations delivering VAD within their facilities, to provision of VAD via established external referral pathways, or to provision of basic information only about government-provided centralised care navigation services who offer information, support, advice and assistance to anyone involved with VAD (called high level “pathways A, B and C” respectively in the Victorian context [3]).

In general, the position statements adopted by palliative care peak bodies have stated that VAD should not be part of palliative care practice as their view is that palliative care should be focused on good quality of life, relief of suffering through holistic assessment and management and never on hastening death [4, 5]. A stance of “studied neutrality”, that is "the careful or premediated practice of being neutral in the dispute about euthanasia” (p. 898) [6] has developed which may be useful to palliative care organizations to explain their position when VAD is being debated within the societal context. However, this stance may no longer provide guidance to facilities, especially those that are non-faith based, to make practical decisions about the services it will offer if VAD is legalized [7].

The literature on how palliative care organisations make decisions about their interface with VAD in jurisdictions where it is legal is limited. Gerson’s 2020 systematic review states that the relationship between palliative care and VAD varies across countries. The relationships are described as co-existing and synergetic (e.g. Belgium), integrated and collaborative (e.g. in some services in Toronto, Canada), or ranging from ambivalent to opposed (e.g. Switzerland)[8]. However Gerson highlights that these relationships are impacted by pre-existing institutional policies and how they actually work in practice are not well described hence there is a need for further research [8]. Canadian researchers have also highlighted a particular need to understand how hospices have been impacted following legalisation of VAD [9].

Studies from Oregon and Washington show that their palliative care services have made a variety of decisions toward VAD [10–12]. Campbell et al., [10] report that although the majority of patients who utilised Oregon’s Death with Dignity law were enrolled in palliative care, most services have boundaries in place to prevent or limit participation with assisted dying legislation. They found that of 55 palliative care services, nine (16%) provided for full participation in VAD, 32 (59%) provided for limited participation, and 14 (25%) did not allow any participation.

Recently published information on VAD implementation focuses on large organisations in which palliative care provision is only one aspect of their activities. These papers discuss the required steps for successful implementation but do not describe in detail how the organisations made their original policy decisions. For example, a multisite cancer care alliance in Seattle simply acknowledged “considerable internal debate” [7] and a tertiary health service in Melbourne stated that their decision to provide a VAD pathway was based upon a survey indicating sufficient staff doctors were willing to perform VAD roles [13]. Similarly, a network of hospitals in Canada described their VAD implementation framework [14], but did not explain how they first explored the VAD participation question, despite reporting that many of their departments were unwilling to be formally associated with VAD provision.

In Australia, the term “palliative care” is generally understood as per the World Health Organisation definition to be “an approach that improves the quality of life of patients and their families who are facing problems associated with life-threatening illness” with a focus on the relief of suffering and care which intends “neither to hasten nor postpone death” [15, 16]. “Hospice” is generally used to describe inpatient units caring solely for such patients, and which are usually designed to feel as homely as possible. They may be separate facilities or rooms within a hospital or residential aged care facility [17]. Community hospices, of which there are few in Australia, are in a unique position in regards to VAD participation decision-making as they are generally smaller, not-for-profit, inpatient palliative care services [18], with independent boards, significant volunteer involvement and reliance on community goodwill for fundraising and philanthropy.

With its 30-year history, the Albany Community Hospice (ACH) in rural WA is the only remaining community-owned hospice operating in the state. It is a fully accredited, eight-bed, free-standing unit with specialist nursing care, managed by a nine-member volunteer Board of Management. Medical care is provided by General Practitioners and a visiting palliative care specialist (author KA). The ACH is a secular entity now but it had significant support from a religious organisation during its foundation years and continues to rely on the local community for financial viability [19]. Prior to legislation, ACH published a position statement that aligned with the peak Australian palliative care body stating that the organization was focused on palliative care and that palliative care did not hasten death nor prolong life [20].

The ACH Board began considering the implications of the new legislation in January 2020. One year later, following a nonunanimous vote, they came to the decision to support full participation with VAD as per the WA VAD Act (see Attachment 1: ACH Voluntary Assisted Dying Position Statement, 18 February 2021). At the time of writing, it is the only non-government inpatient palliative care unit in the State with a policy supporting inpatient access to VAD.

As there is little detailed information available describing the decision-making processes undertaken by palliative care unit executives around VAD access within their facilities, the aim of this study was to describe the experience of the ACH Board as they made policy choices prior to full enactment of VAD legislation.

## Method

### Research team

The research team consisted of the authors of this paper and an experienced interviewer. KA is a palliative medicine academic physician who provides specialist consultation to General Practitioners at ACH and is a medical advisory committee volunteer. She was a representative on the Ministerial Expert Panel which advised the WA government on the original VAD bill [21]. TP has a long career in public health research, especially in community programmes and has no formal links to ACH. KG was the ACH research nurse at the time of the study. RD is a psychologist and health promotion/social marketing academic with no links to ACH. The interviewer (CG) is an independent consultant who worked in local government and community development and has previously facilitated workshops to develop ACH’s strategic plan. The team members were agnostic to the Board’s final policy about VAD implementation.

### Sample

Board members were recruited to the study several months after they began their deliberations through KG attending a Board meeting and via follow-up emails. They were invited to take notes over the coming months to aid future recall of thoughts and events and then to participate in individual interviews conducted by an independent professional interviewer using a semi-structured interview guide. Written informed consent was completed by each participant before commencing. Reminder emails about notetaking were sent approximately two-monthly and suggested that they may document points of stress or difficulty, recent key decisions, issues they “got stuck on”, what helped them move forward, moments of clarity, information they found helpful and their own feelings. Notes were not collected but participants were encouraged to refer to them in the subsequent interview.

The interviews took place within four weeks of the release of the Hospice’s VAD position statement in February 2021 and occurred at the participant’s home or in university rooms. The interviewer documented their impressions following each interview and provided these to the researchers. The interviews were voice-recorded and transcribed and de-identified by a professional transcribing service. All participants approved their own transcript prior to these being analysed by the researchers. Minutes from all Board meetings during that period were reviewed by KG to collect information about whether a VAD discussion occurred at the meeting and whether any VAD-related resources were provided. Table 1 provides a timeline of the decision-making trajectory.

**Table 1:**
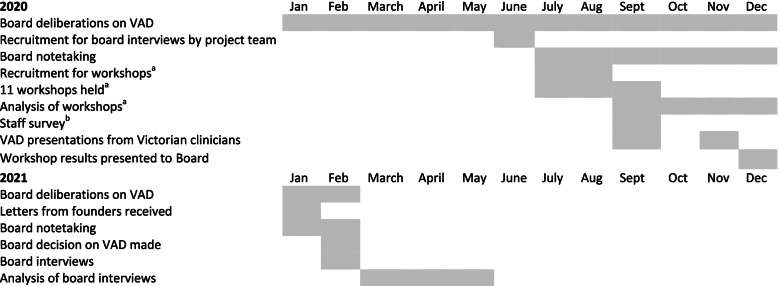
Timeline for the Board deliberations and the research project

^a^the workshops included staff, volunteers and community members and were part of another research project [22]

^b^the staff survey was undertaken, analysed and reported by the Board and did not form part of this project

### Board meeting activities and stakeholder consultations

Voluntary assisted dying was discussed at all ten Board meetings between January 2020 and February 2021 with the aim of informing themselves and consulting stakeholders. The Hospice clinical manager and senior administration officer were present at all meetings to provide clinical information if requested about how the Act may interface with patient care and answer questions but did not otherwise participate in discussions. At the first two meetings, members were provided with a detailed description of the Act and a summary of the minimal existing literature around how other organisations had approached VAD policy decisions and implementation. In May 2020, the Board voted for a community consultation process and confirmed a deadline for a position statement of February 2021, four months before VAD would become legally available.

A series of workshops was conducted by the first three authors between July and September 2020 to explore the expectations of staff, volunteers and members of the public as to how ACH could respond to VAD legislation. Eleven workshops with a total of 63 participants were conducted and the results were presented to the Board in December 2020. The workshops indicated a variety of expectations as to how ACH could respond, although there was general agreement amongst stakeholders that patient-focused care, remaining a “safe place”, transparent policies and awareness of risk to the Hospice should inform the Board’s decision-making [22].

In September 2020, the Board decided to survey staff and volunteers as to their willingness to engage with the VAD process. The anonymous online/paper questionnaire was based on a document provided by the WA Department of Health [23] and was open for completion from mid-November to early January 2021. Questions addressed whether the person agreed that VAD should be available and their willingness to give information, refer patients, participate in assessment or administration and be present in the room or facility at the time of VAD occurring. Board minutes show that 26 nursing and administrative staff (response rate = 87%), nine volunteers (response rate = 45%) and 21 doctors (response rate = 35%) completed the survey. Most staff indicated they were willing to engage in some part of the VAD process within the scope of their professional role, with eight of the doctors being willing to fulfill roles as practitioners under the Act (unpublished data).

During this period, two speakers from Victoria were invited to present their experiences to Board meetings: a manager from a hospital providing VAD (September 2020) and a medical ethicist working in specialist palliative care (November 2020). Several Board members also asked for out-of-session meetings with clinical staff to discuss death and dying in more detail.

A month prior to the final vote in February 2021, scenarios of different levels of organisational facilitation of VAD were presented to the Board for consideration and discussion. At this time, the Chair of the Board approached two of the founders of ACH asking for letters of advice to the Board regarding the VAD decision, which were read at the final meeting. The usual Board process of majority support (rather than unanimous) carried the vote. The subsequent position statement that ACH would “assist and support those patients who feel they are suffering intolerably, and who wish to access Voluntary Assisted Dying” was shared with staff to inform them at two dedicated forums prior to being uploaded to the organisation’s website (see Attachment 1 for position statement).

### Data analysis

Data were analysed using conventional qualitative content analysis method, chosen as existing theory and research literature is limited, and the method allows themes to flow from the data. [24]. The researchers initially repeatedly read the interview transcripts and interviewer notes in entirety to gain a sense of the overall picture, then re-read to develop codes relating to various ideas, actions or events. Team members then independently assigned these codes to the transcript data, highlighting key words, sentences and paragraphs. The coded transcripts were compared, and discrepancies discussed until agreement on broad themes was reached. These themes were shared with participants prior to finalization of this paper who agreed with the results presented.

### Ethics

Ethics approval was granted through the University of Western Australia Human Research Ethics Committee (2019/RA/4/20/6324).

## Results

### Participants

Seven of the nine Board members consented to participation. The reason for non-participation was not sought from the remaining two. Interviews ranged from 28 to 47 min (mean = 38 min) and five participants referred to their notes. Participants had been volunteers on the Board for one to five years (mean = 3) and held portfolios in medical advisory (*n* = 1), finance and risk management (*n* = 4), liaison with the charitable second-hand goods store (“op shop”) (*n* = 1), fundraising (*n* = 1) and business development (*n* = 2). Six of the seven board members interviewed were female. Two participants had a background in healthcare (psychology, medicine). Other demographic details were not collected to preserve anonymity. Extensive use is made of participants’ verbatims in the below analyses with participants referred to by number (#1–7) to protect their confidentiality and reflect the range of responses across different individuals.

### Major findings

Overall, participants described the decision-making undertaken by the Board as ‘a journey’. Hence, we present the results in terms of the stages of this journey and the themes within. Key facilitators of a successful process, the personal impact of the journey on participants, and their recommendations to other boards facing this (or a similar) decision are presented.

### Beginning the journey: Starting from a personal view

As expected, when people are faced with making a decision about an issue, their existing beliefs, attitudes and past experience with respect to that issue come to mind [25]. Several participants discussed the influence of personal or formative experiences on how they initially thought about VAD, including their faith, family upbringing, professional background and personal interests.

Some participants already held a positive attitude towards VAD: “*I personally am very pro voluntary assisted dying*” (#3); “*I am very definite that I want VAD for me*” (#5). Others held neutral or ambivalent views being “*still a bit on the fence about it personally*” (#7), and at least one felt it was against their own ethical framework: “*I always had a view that VAD was not palliative care, from the view of being professional and a personal view being a Christian*” (#1).

For several, the discussions triggered recall of friends or family they believed could have benefited from VAD had it been available at the time: “*I had a friend who when dying would say to his wife and myself,’I just want the pill, I just want to die’*” (#2). Others referred to their personal values such as “*a fundamental belief in a person’s right to choose*” (#6), compassion, and a desire to prevent suffering.

### Moving from a personal view to a Hospice perspective

Although participants’ early thoughts were based on their personal views and experiences, a key facilitator of the decision process was then framing the VAD implementation decision as not about personal beliefs and feelings, but about “*what is the right decision for the Hospice?*” (#3). This was expressed in a number of ways: “*having a personal view doesn’t frame the way that you, as a Board member, are going to actually work in it – our patients are going to choose, patients are going to think about it*” (#1); “*(I needed to) dissociate what I think personally from the separate issue about what the Hospice needed to do about VAD*” (#7); and “(*I needed to) listen to all the arguments for and against because I was making a decision that wasn’t actually for me. I knew as a Board member I had to consider the religious side for the people that are religious*” (#5).

These reflections then led to the next stage of the journey—the consideration of whether “*[VAD] fits inside the notion of palliative care or is it something different to palliative care?*” (#7).

### Exploring whether VAD can be part of end-of-life care

In coming to a decision, participants realized they needed to articulate what the term “palliative care” meant. This resulted in most coming to the view that VAD was not part of palliative care, given their understanding that palliative care did not include helping to hasten death. However, they did agree that “*voluntary assisted dying can be part of end-of-life care*” (#1). This consideration was voiced by one as that “*our role at Hospice was about loving kindness and about caring for people. It wasn’t just about palliative care, but it was about having a passion for people in the end-stage of life and that’s what voluntary assisted dying has been about the whole way through—being compassionate to the people in their choices at their end-stage of life*” (#2).

Despite accepting the definition of palliative care, some saw little real difference between previously accepted practice and VAD: “*why do we suddenly draw a line there?*” (#4); “*why do we even have to make a decision?*” (#5). These participants felt there was little difference between VAD and “*someone deciding to ask for treatment to be stopped, to refusing food and water, terminal sedation*” (#4), and when “*people are terminally sedated in Hospice and in the end the person dies quietly and pain free*” (#5).

Given this general acceptance of VAD as part of end-of-life care and hence something that could be supported by the Hospice, the next stage of the journey involved an assessment of risks to the organisation in making a decision either way.

### Consideration of risks to the hospice

Participants considered there were potential risks to ACH’s reputation and viability given that VAD is seen as “*controversial*” (#7) and that “*no matter what we did there will be people who are not going to be happy*” (#1). However, most risks were discussed in the context of the consequences of the Hospice accepting rather than rejecting VAD participation.

As an organisation highly reliant on fundraising, there were concerns about a “*potential impact on fundraising and donations*” (#1, 3, 4, 6), as well as the possibility of “*people with placards protesting outside of Hospice*” (#2) or “*taking out their negativity on individual Board members*” (#4). Participants were also concerned that there may be unintended negative consequences of endorsing VAD as “*going down the VAD route could actually scare people away from even taking up palliative care, actually frightening people from coming to Hospice*” (#7). Other risks noted included: being isolated—“*an orphan—a private hospital that will accept people for VAD where there may be no others in the state*” (#2); staff or Board member resignations; lower staff wellbeing; impacts on family; and potential additional stress during the COVID situation.

Given an overall assessment that there could be risks, the next journey stage involved considering these risks in the context of the hospice’s underlying values with respect to commitments to patients, and hence a common platform on which to vote.

### Arriving at a common platform to vote on

The participants noted that “*patient choice*” (#1, 2, 4, 6), “*respecting our guests’ wishes*” (#2), and the desire to “*support [people] to the max to ensure what they want is what can be achieved for them*” (#7) were the main values that enabled them to make a decision about VAD access within ACH. That is, the decision to support patients wishing to access VAD was not only consistent with the view that VAD could be part of end-of-life care but was also based on the members coming to a shared view with respect to the Hospice’s values. Once this decision was made, several participants expressed a belief that the identified clinical and team-based risks and challenges could be managed by the “*strong and mature*” (#6) senior staff through a VAD implementation committee that was formed immediately after the position statement was released:

“I have the greatest confidence that the staff just have the ability and the will to make this work and they’re all really prepared… we will see all those policies and procedures” (#3).

There was a hope that there “*would be a sensible absorption of the decision without too much flack*” (#7) however others highlighted that the Board would need to be proactive in monitoring staff wellbeing, resignations, public comment and fundraising revenue and be prepared to respond.

### Factors facilitating a safe decision-making journey

A notable feature of the Board’s decision-making process in this controversial area was an absence of acrimony and a common desire to put personal feelings aside and make the decision that was best for the organization. It was also reported as vital that the usual Board business would proceed as normal and that clinical or procedural issues around VAD implementation would be explored separately. Whilst the members’ approach to the issue and their commitment to their roles contributed to the process proceeding amicably to a nonunanimous decision, two procedural factors—having sufficient time and the provision of substantial relevant information and feedback from a variety of sources—appear to have significantly facilitated the outcome.

#### Sufficient time

The benefits of “*a lengthy period of time, so that people had a chance to sort of sit with the ideas over some months*” (#7) was referred to by all participants as a lesson for other executive teams facing such decisions. These benefits included that enough time made it “*very stress-less*” (#5), prevented people feeling “*overwhelmed*” (#7) or “*panicky*” (#2), and that the decision-making “*felt fluid*” (#1), “*naturally evolved*” (#2) and “*happened organically*” (#6). Adequate time also allowed decision-making to proceed through several phases: “*an exploration phase where people were getting more information, beginning to express opinions*” (#4); “*thinking time, just sort of sitting with the concept and kind of letting your brain get used to the notions*” (#7); and time for opinions to vary and be exchanged back and forth.

#### Resources

Several resources were highlighted as useful, not only with respect to providing basic information, but also with respect to helping resolve differences of opinion: “*it was always the information and knowledge that resolved it*” (#4). Useful resources included “*understanding the legislation in a fair amount of detail*” (#6), “*the [community] research that we got given back to us*” (#1), journal articles, staff and volunteer survey results, and information about experiences in other jurisdictions especially Victoria and Canada. The scenarios of different levels of participation in VAD were also considered useful and solidified some participants’ concerns about the requirement to transfer, and hence “*abandoning*” (#2) a patient, if VAD was not allowed within the facility.

#### Workshops

Although it was noted that “*a single community view one way or the other …would have made life easier*” (#7), information gained from the public workshops was described as a “*critical*” (#6) and especially with respect to others having “*an appreciation of the complexity of the situation*” (#4). In that sense, findings from community consultation was a reassurance to the Board that “*if we did choose to go down the VAD line, the community would understand that we had been through a pretty rigorous process*” (#6).

#### Staff and volunteer surveys

Several participants reported that results from the staff and volunteer surveys showing “*that the critical mass [is] supportive*” (#3) was “*the turning point*” (#6) in their decision-making. Although one participant commented that it was “*annoying*” (#6) that this information came late in deliberations, this participant also reflected that “*after pondering that for a while I think it was good that we didn’t get it till the end. I think making me go through a process of really understanding everything before I got their information was a better option because it would’ve swayed me earlier on. I probably would’ve come to the same conclusion, but I think I’ve felt more comfortable having been through that process*”. (#6).

#### Letter from one of the founders of ACH

One significant factor for several participants was a letter from one of the founders of ACH, a person held in high regard by Board members, which referred to an acceptable decision being one that would “*be doing our best to support the needs of the Hospice’s patients*” (#7). Given this person’s status and religious perspective, this letter was perceived as giving “*permission to make whatever we collectively thought was the right decision*” (#6). It was described as “*important*” (#4), “*a turning point*” (#5), and “*a comfort*” (#3).

#### Other resources

Other items and activities reported to be useful included “*videos prepared by the [State health department] implementation committee*” (#4), “*individual conversations with the Hospice manager from a clinical perspective to clarify some things*” (#6), and video-conferenced discussions with practitioners from Victoria. The latter were key events for some as “*it became apparent to a certain extent that it was OK to be conflicted about [your] position*” (#3) and that there was great value in “*hearing from others who were actually engaged in the process but not necessarily strongly for or against*” (#4).

These resources allowed participants to “*develop our opinion – our ideas*” (#2) and be reassured “*that this decision didn’t have to be based on feelings and emotions. It was a well thought-out, well-researched and methodical decision*” (#1).

#### Board culture

A “*completely professional*” (#3) Board culture, along with a “*sense of we’re all in this together and could be completely open*” (#3) were considered important facilitators for a successful decision-making process: The Board meetings were described as “*a very supportive environment for people to discuss very difficult issues in a …respectful setting*” (#3). The role of the Chair and Board procedures were seen as important to “*allow people to have input in circumstances where they feel safe to be able to say what they think without any pushback*” (#7), and where it was understood it was acceptable to “*just abstain…. go out the room—no one’s going to mind*” (#2). In essence, there was an atmosphere of trust amongst Board members: “*The experience of the honesty that the other board members brought to the table I found an incredibly positive thing and sort of reinforcing that everyone is giving all they had to this discussion*” (#7).

Participants stated it made them feel more comfortable when the final decision was made to allow VAD in the facility, that they had been “*inclusive and transparent*” (#1), both with respect to the Board discussions and the consultation component: “*We needed to bring people who work here along with us, and they had to be allowed to feel they had been heard and that they had been part of the process of thinking about this*” (#7). A further support to decision-making for some was the process of taking notes as part of their participation in this research study: “*doing the diary really made you sit down and focus on what you were doing—the process of writing provided me with a level of clarity” (#6); “we know the value of reflection—it changes people*” (#4).

### Personal impact of the journey

Overall, the responsibility of decision-making was experienced as “*huge*” (#2, 3, 5, 7). All participants reported that the process took “*a toll*” (#1) on themselves. Part of this was due to the “*enormous amount of work*” (#3) involved, especially with other COVID-related impacts during that period and as a number were working full-time. In addition to experiencing “*stress*” (#2, 7), there were reports of feeling “*conflicted*” (#7), experiencing “*a constant thought about what it means to the specialty [palliative care]*” (#1), being in a “*mental dilemma*” (#3), feeling “*surprised that I became emotional about it*” (#3), including one who felt “*angry… and just shocked*” (#4).

On the other hand, participants described several personal benefits and positive experiences of the process: “*I [am] very lucky because we had the opportunities to think about our dying and to discuss these things without fear or judgement, whereas most people in the community don't have that*” (#2). One mentioned that it was “… *a massive learning curve …. but incredibly interesting*” (#6), whilst others reported feeling “*proud*” (#7) of their work and experiencing a sense of “*understanding*” (#7) and “*graciousness and goodwill*” (#3) towards themselves from various stakeholders.

### Participants’ recommendations to other boards facing this decision

Reflecting the importance of having sufficient time, being provided with substantial information and resources, engaging in consultation with the community, staff and volunteers and the Board culture of cooperation, the participants came to two overall recommendations (or “lessons”) for other executive teams faced with this decision:

*“Give yourself enough time to allow people to be appropriately informed about what’s involved, appropriately informed about the experience of other jurisdictions that have done it so that people have a sense of how it actually works. People on the board need to have an adequate amount of time to process their own thinking about it. This is a decision that can’t be done in a rush. It does require the balancing of legal concepts, moral concepts, sometimes religious concepts.”* (#7)

*“Learn as much as you can: listen to your community and your stakeholders - all of them; engaging as much support as you can.*” (#2)

## Discussion

This research recorded the journey of a volunteer Board of Management of a rural community-owned and operated hospice making a policy decision around VAD implementation. It describes how the process was experienced by participants, most of whom had no background in healthcare and highlights facilitators of an effective outcome. As the final position was to allow full participation with the VAD legislation within the facility, it is a rare exploration of the conditions that resulted in organizational “conscientious participation” [26], whereas much of the literature is around organizational conscientious objection or the factors that impact on individuals’ decision to participate in VAD provision. The data analysis described an abstract phenomenon on which existing theory is limited (VAD decision-making as a journey) as well as simple themes around useful factors that could be considered by other Boards.

The importance of enough time and information to help them move forward and reach clarity was emphasized by participants. It has been shown elsewhere that developing a VAD position statement takes time even in well-resourced and expert organizations; for example, the Board of the International Association for Hospice and Palliative Care required many months to clarify their method for development of their statement and to identify key resources, then further months of discussions between members to reach a decision [4].

A key framing for the ACH Board to allow participation in VAD was that it is a choice at end-of-life outside of usual palliative care practice, but within the services that could be offered by a hospice. This reflects the pluralist approach from Belgium where VAD is relatively uncontentiously positioned as another option at the end of a palliative care pathway due to the country’s tradition of liberalism, co-development of both services, high profile caregivers being active in both domains, explicit policy direction and release of shared guidelines [27, 28]. Dierickx’s 2018 study of the interface of palliative care and euthanasia in Belgium confirms the ongoing reality of this approach by demonstrating that palliative care practitioners are frequently involved in consultation about and administration of euthanasia [29]. It is also consistent with calls from New Zealand for facilities (called “dignity trust havens”) where people would have the option of both palliative care and VAD at the end-of-life [30].

The negative impact of inpatient palliative care units not providing a VAD service has been raised by Waran and William who present the case of a patient within a Victorian unit who requested access to VAD [31]. They discuss challenges in supporting patients who want to make a VAD choice in this situation especially with respect to the need to transfer patients to another facility, the desire to keep palliative care as a distinct and separate practice, and concern about the diversion of limited healthcare resource allocation from palliative care to VAD. Such issues were raised by participants in this study as they serve in a community where there are no other inpatient unit options.

Although Booth et al.’s., article on the implementation of a VAD program in a major public health service in Melbourne did not discuss the process of making a decision in detail [13], once made, implementation logistics were informed by similar issues expressed by participants in this study, including patient-centeredness and mitigation of organisational risk. Their reported requirement for conversations with stakeholders to be sensitive, honest and respectful, including with the community and with those of opposing views [13] was similar to this study’s findings. It is also of note that the willingness of a small cohort of doctors to be involved was a key piece of information for the executive in both cases.

Board members were clearly aware of the significance of the decision to be made and the uncertainty of its impact, which resulted in times of stress, but also some personal growth. Making a decision that would result in high quality end-of-life care, support families, have stakeholder acceptance and ensure organisational safety was experienced as difficult due to the ambiguity of community expectations and complex relationships between stakeholders including patients, their loved ones, clinicians, state government funders and community fundraisers. However, the provision of stepped information over a prolonged period allowed for a concrete outcome.

Since legalisation, many hospices in Canada have declined participation in VAD (referred to there as Medical Assistance in Dying [9]). For some this was on faith-based grounds, but for others, considerations for non-participation included a lack of institutional capacity and expertise, the potential of a contradiction with palliative care and a concern not to conflate VAD with palliative care in the public consciousness [32]. These concerns were also present in this study but were overcome by a focus on patient choice and delivery of a wanted service.

### Strengths and limitations

The request to keep notes during the process may have increased participants’ attention to the topic as they also knew they would be questioned later. However, keeping notes also resulted in most having a record to refer to rather than relying on memory. This study did not include executive teams from other hospices in WA and hence reflects a singular experience which may not be generalizable to other organizations.

## Conclusion

This qualitative study adds valuable information to the developing narrative on how palliative care organizations interface with VAD participation decisions once it becomes legal within their jurisdiction. The journey described by this group of volunteers on a community-owned hospice Board also highlights facilitators of a successful outcome, including adequate time, useful resources, sound Board processes and a trusting culture. Their experiences may provide guidance to other palliative care organizations as they face similar decisions.

## Supplementary Information

**Additional file 1: Attachment 1.** AlbanyCommunity Hospice Voluntary Assisted Dying Position Statement.

**Additional file 2: Attachment 2.** Semi-structured Interview Guide for Board Members.

## Data Availability

The datasets generated and analysed during the current study are not publicly available due to the small number of board members interviewed and the confidential nature of the interviews but are available from the corresponding author on reasonable request.

## Abbreviations

ACH: Albany Community Hospice
VAD: Voluntary Assisted Dying
WA: Western Australia
