# Supplementary material for: “Respecting our patients’ choices”: making the organizational decision to participate in voluntary assisted dying provision: findings from semi-structured interviews with a rural community hospice board of management
Source: BMC Palliat Care. 2022 Sep 16;21:161. doi: 10.1186/s12904-022-01051-x (PMC9482306; doi:10.1186/s12904-022-01051-x)
Supplement: Supplementary file 1 — Additional file 1: Attachment 1. AlbanyCommunity Hospice Voluntary Assisted Dying Position Statement. [file 12904_2022_1051_MOESM1_ESM.docx]

**Attachment 1: Albany Community Hospice Voluntary Assisted Dying Position Statement**


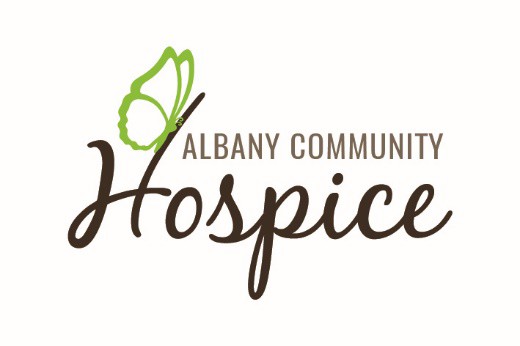
 ALBANY COMMUNITY HOSPICE POSITION STATEMENT

**Voluntary Assisted Dying Position Statement**

It is acknowledged that there is a diversity of opinion in public and healthcare communities about Voluntary Assisted Dying in end-of-life care.

Albany Community Hospice (ACH) has prepared this position statement to inform the community of the ACH Board’s recent decision on how ACH will respond to the new Voluntary Assisted Dying legislation that is effective in WA from July 2021.

The newly adopted law for Western Australia has now introduced a new mechanism that allows competent terminally ill persons, whose death is approaching and whose suffering is intolerable to them, to be assisted to die if they wish. This will be by the taking of designated lethal medication by the person themselves where they are physically able to do so. If they are not, then they may request assistance by injection from their doctor.

The Board has considered the appropriate response to the legislation carefully and respectfully over many months. It has consulted widely within the community and with the doctors, staff and volunteers who work at ACH in order to inform its deliberations. It has considered information about the Victorian experience given its similar legislation.

Having considered the feedback given in community consultation and from staff and volunteers, the Board has taken the view that the focus must be on providing choice to those dying in our facility. It has been decided that, in addition to continuing to provide expert palliative care ACH should also support competent persons who meet the legislated criteria and who choose, in consultation with their doctor, to adopt this new end-of-life path and elect Voluntary Assisted Dying. Accordingly, from July 2021, eligible patients who chose Voluntary Assisted Dying will be permitted to access it whilst residing at ACH.

In making this decision, the Board aimed to stay true to ACH’s value of compassion and our mission statement:

*‘Albany Community Hospice is a leader in the provision of expert palliative care, education and support so all members of the community can experience death with grace in a manner and place of their choice’*

Given the change in the law, and the Hospice mission’s mandate, it is incumbent upon ACH to assist and support those patients who feel they are suffering intolerably, and who wish to access Voluntary Assisted Dying thereby to end their lives safely and in a safe and comfortable environment in the care of people they trust.

As we have done since being established in 1990, ACH aims to support any patient with a life limiting condition by providing access to evidence-based holistic end-of-life care. The specialist palliative care we provide remains our main focus, with the intent to relieve suffering and improve quality of life in a compassionate, dignified and respectful way. In the main, this will be by the provision of expert palliative care, neither hastening nor prolonging life, and respecting at all times a patient’s rights to refuse life sustaining treatments and to have effective symptom relief and pain management.

The specialist palliative care we provide remains our main focus, however the Board believes that Voluntary Assisted Dying has a place alongside palliative care as a newly available end-of- life option for patients who choose it. Once a competent person has reached the considered view that their suffering is intolerable and they wish it to end, then that view, if it is clear and enduring, needs to be respected and the person supported if they then choose, in discussion with their doctor, to access Voluntary Assisted Dying.

Support will include ongoing expert medical and nursing care, and personal support for the patient and their family. This will include assistance as is requested with the required medical assessments and permit application for Voluntary Assisted Dying together with the planning for the end of life. It will also continue to include a compassionate response to distress and fear, and a safe and respectful environment for patients to talk about their end-of-life wishes without judgement. ACH strives to be inclusive and considerate of the needs of all cultures including the cultural needs of Aboriginal and Torres Strait Islander Peoples and those who may not have English as their first language. We continue to strive to do our best to be inclusive of all people in our community.

ACH places great importance on patients feeling safe. Fundamental to the Voluntary Assisted Dying process is that it remains voluntary and free from coercion. The patient can withdraw or revoke their involvement at any stage. Those patients who, for religious, cultural or other reason, are opposed to VAD may be assured of our commitment to the provision of continuous expert palliative care to them.

The legislation allows health practitioners to conscientiously object to involvement in Voluntary Assisted Dying and this right will be respectfully accorded to all our staff and volunteers without judgement. The ability of ACH to support access to Voluntary Assisted Dying by patients will at all times be contingent on the numbers of trained Health Care professionals including GPs, nurses and support staff being sufficient to staff the ACH on-call team for Voluntary Assisted Dying purposes. Given the feedback received, the Board has every reason to expect that this will be able to be done at the present time.

The Board proposes to review the operation of Voluntary Assisted Dying at ACH at the end of 2022 after the legislation has been in operation for 18months in order to take account the evidential experience of the patients and staff at ACH, the evolving community perceptions and the ongoing development of our procedures and practice of VAD.

ACH has served the Great Southern for thirty years thanks to the substantial goodwill of and contributions (both financial and non-financial) from our community.

The Board trusts that the community will accept that this decision has not been made lightly and that our focus always has been and continues to be the needs and wishes of our patients. It is hoped that the community will understand and accept the Board’s decision in this light despite the ethical dilemmas that, it is acknowledged, the subject will present to some. Please be assured that ACH continues to be guided by its founding principle of loving kindness.

Any queries on this position statement should be directed to the Board Chairperson at [Chair@albanyhospice.org.au](mailto:Chair@albanyhospice.org.au)

The Board encourages members of the community to read the explanatory information on Voluntary Assisted Dying being provided from time to time by the state health department <https://ww2.health.wa.gov.au/voluntaryassisteddying>and to take advantage of such other community information opportunities on the topic as the department and others provide over time.

**18 February 2021**


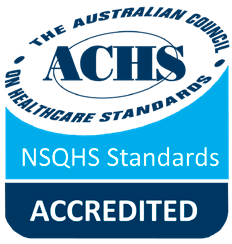
**Endorsed by:** Board of Management – Albany Community Hospice

**Albany Community Hospice**
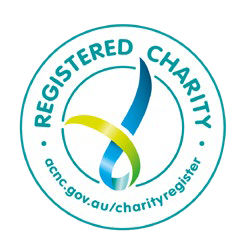
Diprose Crescent, Albany WA 6330 | PO Box 5210, Albany WA 6332
Phone: (08) 9892 2456 | Email: [admin@albanyhospice.org.au](mailto:admin@albanyhospice.org.au) | [www.albanyhospice.org.au](http://www.albanyhospice.org.au/) ABN: 60 467 001 291
