# Supplementary material for: “Respecting our patients’ choices”: making the organizational decision to participate in voluntary assisted dying provision: findings from semi-structured interviews with a rural community hospice board of management
Source: BMC Palliat Care. 2022 Sep 16;21:161. doi: 10.1186/s12904-022-01051-x (PMC9482306; doi:10.1186/s12904-022-01051-x)
Supplement: Supplementary file 2 — Additional file 2: Attachment 2. Semi-structured Interview Guide for Board Members. [file 12904_2022_1051_MOESM2_ESM.docx]

**Attachment 2: Semi-structured Interview Guide for Board Members**

**Semi-structured interview guide for Board members**

Welcome and thank you for being available for this interview

Introduction to interview:

As Board member you will have been part of the deliberations on how Hospice will respond to the enactment of The Voluntary Assisted Dying Act 2019 in June of this year.

There is minimal current information on the interface between palliative care services and voluntary assisted dying in jurisdictions where the latter is legal to guide services in how to approach these new and challenging organisational conversations. It has been said that there is a “pressing need” for further research in this area.

In this critical short phase between passage of the legislation and enactment, this interview aims to explore the key events and processes that occurred as part of the Board’s deliberations regarding enactment of the legislation within WA. Each interview will be voice recorded and transcribed by a professional service. All transcriptions will be de-identified before being reviewed by yourself, when it will be possible to make changes.

To start, I will ask you some general questions about Board membership:

- How long have you been a member of the Board?
- Do you hold any portfolios or areas of special interest on the Board?
- Can you tell us a little bit about why you joined the Board?

*Please feel free to review your notes as you need to.*

I’d like hear your personal reflections on your experience as a member of a Board making decisions about voluntary assisted dying.

- Did you note any points of stress, difficulty or feelings of confusion?
- What were the key decisions points that you recall?
- Were there any points that you got ‘stuck’ on?
- What helped you move forward when you did get stuck?
- Were there moments of clarity or understanding or changes in perception that you noticed within yourself?
- Were you comfortable in your role as a Board member during this time?
- Were there any personal beliefs that may have influenced your decision making?
- Were there any personal experiences that may have influenced your decision making?

Now, thinking about how the Board worked together to come to some resolutions:

- What do you think were the main factors that helped the Board make their decision?
  What types of information helped?
  Were there any particular events that you think may have influenced the Board?
  Do you recall if there was an increase in the public debate related to voluntary assisted dying? Do you think that this may have influenced the Board in their decision making?
- How do you recall the Board reacting when given the focus group feedback? Do you think that the Board were surprised with the results?
- How did the Board incorporate what the community, staff and volunteers were saying?
- Do you see any challenges with carrying out the decisions the Board will make?
- What advice would you give another Board facing similar organisational decisions?

Any further comments?

Thank you
